# Rhinovirus C targets ciliated airway epithelial cells

**DOI:** 10.1186/s12931-017-0567-0

**Published:** 2017-05-04

**Authors:** Theodor F. Griggs, Yury A. Bochkov, Sarmila Basnet, Thomas R. Pasic, Rebecca A. Brockman-Schneider, Ann C. Palmenberg, James E. Gern

**Affiliations:** 1 Department of Pediatrics, School of Medicine and Public Health, CSC K4/945, 600 Highland Ave, Madison, 53792 WI USA; 2 Cellular & Molecular Pathology Graduate Program, Madison, WI USA; 3 Medical Scientist Training Program, Madison, WI USA; 4 Department of Surgery, School of Medicine and Public Health, Madison, WI USA; 5 Institute for Molecular Virology, University of Wisconsin, Madison, WI USA

**Keywords:** Rhinovirus C, Air-liquid interface, Ciliated cells, CDHR3, Bronchial epithelium

## Abstract

**Background:**

The Rhinovirus C (RV-C), first identified in 2006, produce high symptom burdens in children and asthmatics, however, their primary target host cell in the airways remains unknown. Our primary hypotheses were that RV-C target ciliated airway epithelial cells (AECs), and that cell specificity is determined by restricted and high expression of the only known RV-C cell-entry factor, cadherin related family member 3 (CDHR3).

**Methods:**

RV-C15 (C15) infection in differentiated human bronchial epithelial cell (HBEC) cultures was assessed using immunofluorescent and time-lapse epifluorescent imaging. Morphology of C15-infected differentiated AECs was assessed by immunohistochemistry.

**Results:**

C15 produced a scattered pattern of infection, and infected cells were shed from the epithelium. The percentage of cells infected with C15 varied from 1.4 to 14.7% depending on cell culture conditions. Infected cells had increased staining for markers of ciliated cells (acetylated-alpha-tubulin [aat], *p* < 0.001) but not markers of goblet cells (wheat germ agglutinin or Muc5AC, *p* = ns). CDHR3 expression was increased on ciliated epithelial cells, but not other epithelial cells (*p* < 0.01). C15 infection caused a 27.4% reduction of ciliated cells expressing CDHR3 (*p* < 0.01). During differentiation of AECs, CDHR3 expression progressively increased and correlated with both RV-C binding and replication.

**Conclusions:**

The RV-C only replicate in ciliated AECs in vitro, leading to infected cell shedding. CDHR3 expression positively correlates with RV-C binding and replication, and is largely confined to ciliated AECs. Our data imply that factors regulating differentiation and CDHR3 production may be important determinants of RV-C illness severity.

**Electronic supplementary material:**

The online version of this article (doi:10.1186/s12931-017-0567-0) contains supplementary material, which is available to authorized users.

## Background

Non-influenza viral respiratory illnesses cost the US an estimated $39.5 billion annually from both healthcare costs and loss of productivity [1]. Rhinovirus (RV) is the most common cause of viral upper respiratory illness resulting in rhinitis, sinusitis, pharyngitis, or otitis media, and can lead to the development of bacterial superinfections [2–7]. While most individuals only experience mild symptoms during a RV infection, children, the elderly, the immunosuppressed, and those with asthma, COPD, or cystic fibrosis are predisposed to lower respiratory tract illnesses including wheezing, asthma exacerbations, and respiratory distress that can often result in hospitalization [8–14]. RV has also been implicated in the initiation of asthma, as the presence of wheeze during acute RV infection in the first few years of life is strongly correlated with the development of asthma later in life [15–19]. Three RV species (A, B and C) have been identified, and recent studies have established a link between RV species and illness severity [14, 20]. The RV-C, discovered in 2006, appear to instigate more severe upper and lower respiratory tract symptoms in infants and children under 5 years of age [14, 20]. In an effort to explain this phenomenon, Nakagome et al. inoculated airway epithelial cells with multiple members of each RV species, and observed the highest levels of viral replication, cytotoxicity, and release of inflammatory cytokines following inoculation with either RV-A or C [21].

Virus interactions with the host epithelium are an important determinant of illness severity, and the initial step in this interplay is binding to the target cell via a cell entry receptor. Consider, for example, that cellular retargeting of avian influenza virus in human tissue from ciliated to secretory cells is necessary to produce severe and clinically apparent disease, and also that a broadening of the human coronavirus tropism to alveolar pneumocytes contributed to the Severe Acute Respiratory Syndrome and Middle East Respiratory Syndrome pandemics [22–30]. For RVs, the cellular targets of the RV-A and B have been studied with varying results [31–35]. RV-B14 replication and expression of the major group RV receptor ICAM-1 have been identified in non-ciliated cells derived from the palatine tonsils, and high levels of RV-A16 (A16) replication were noted in cultures with mucus cell metaplasia, which can occur in asthma or COPD [31, 32, 34]. Significant ICAM-1 expression and A16 binding were also observed on basal epithelial cells [33, 36]. More recently, Jakiela et al. contradicted previous studies by identifying ciliated cells as the primary target for A16, and further indicated mucus cell metaplasia as a protective factor against A16 infection [34, 35]. Regarding the RV-C, Bochkov et al. demonstrated the presence of viral RNA in sinus mucosal tissue organ cultures, and subsequently identified cadherin-related family member 3 (CDHR3) as the first and only known RV-C entry factor. However, the specific host cell for the RV-C remains unknown [37, 38].

We therefore performed a series of experiments to identify the cellular target of the RV-C. Our primary hypotheses were that the RV-C target ciliated airway epithelial cells (AECs), and that infectivity was restricted to cells expressing CDHR3.

## Methods

### Viruses

RV-C15 (C15) and RV-C15-GFP suspensions were prepared from the pC15-Rz and pC15-GFP plasmids by reverse genetics as previously described [21, 37–39].

### Cell culture

Primary human bronchial epithelial cells (HBECs) were extracted from trachea or main stem bronchus trimmings of healthy donor lungs that were transplanted and provided by the University of Wisconsin Department of Surgery, Division of Transplantation and grown by the air–liquid interface (ALI) culture method as described previously [40, 41]. For some experiments, we used a second culture medium (PneumaCult™, Stem Cell Technologies, Vancouver, BC, Canada) after preliminary experiments demonstrated improved differentiation and susceptibility to RV-C infection. CDHR3 expression was quantified by PCR as previously described [38].

### ALI infections

Basal medium was aspirated from 30–50 day-old ALI cultures that were then washed apically 5x and incubated with 100 μl of apical BEGM containing 1 x 10^7^ (2 x 10^6^ for gene expression and western blot analyses) plaque forming unit equivalents (PFUe) of C15 (3.5 h, 34 °C). The inoculum was then aspirated, basal ALI media replenished, and cultures were incubated for an additional 14.5 h.

### Immunofluorescence

Following overnight incubation with C15, ALI cultures were washed 3x apically and basally with PBS and fixed with ice cold 4% PFA (15 min, RT). Membrane inserts were cut in half and removed from transwell plates and placed into a 24-well plate for staining. Cells were permeabilized in 0.3% (v/v) Triton-X100 (10 min, RT), blocked in 0.5% nonfat dry milk in PBST (1 h, RT), and incubated in primary antibody (1:200 in blocking buffer, 1 h, RT), secondary antibody (1:250 in PBST, 1 h, RT) and Syto-13 (0.5uM, 10 min, RT, Life Technologies, Grand Island, NY). Inserts were washed in between staining with PBST, and mounted with ProLong® Gold Antifade Reagent (Life Technologies, Grand Island, NY). Cells were imaged on an Olympus 1X71 fluorescent microscope with a Q imaging Retiga2000R camera, a Nikon Eclipse T*i* fluorescent microscope, or Nikon C1 laser scanning confocal microscope (Chiyoda, Tokyo, Japan) with a 60x oil immersion objective. Analysis of digitized images was performed with FIJI/Image J version 1.49 h (NIH, Bethesda, MD).

### Immunohistochemistry

Differentiated cell cultures were fixed with 10% normal-buffer formalin, and embedded in paraffin (University of Wisconsin Histology Lab, Madison, WI). Five μm sections were adhered to slides which were deparaffinized and then rehydrated. For antigen retrieval, slides were incubated with proteinase K (40 μg/mL in PBS, 10 min, 37 °C). Peroxidases were blocked (5 min, RT) with Peroxidazed 1 (Biocare Medical, Concord, CA). Slides were blocked (3% FBS, 2% goat serum, 0.2% Tween-20, 1.25% Human BD Fc Block™, 1 h, RT), incubated (1:200 in blocking buffer, 2 h, RT) with anti-C15-VP2 mouse monoclonal antibody (kindly provided by MedImmune Inc., Gaithersberg MD), Mach 4 Universal Probe and then Polymer (15 min, RT each, Biocare Medical, Concord, CA), Betazoid DAB (5 min, RT, Biocare Medical, Concord, CA), and counterstained with CAT hematoxylin or eosin (30s, RT, Biocare Medical, Concord, CA). Images from labeled slides were acquired and analyzed using an Olympus BX60 light microscope with DP Controller and Manager software (Shinjuku-ku, Tokyo, Japan).

### Flow cytometry

Basal medium was removed from each well, followed by three washes in calcium-and-magnesium-free-PBS (CMF-PBS) apically, and basally. Cells were trypsinized (200 μl apical, 800 μl basal, 8 min, 37 °C) and suspended vigorously with FBS (200 μl, apical), followed by centrifugation (700 x g, 5 min) and decanting. Samples were treated with 0.1% (v/v) Ghost Dye™ Red 780 (Tonbo Biosciences, San Diego, CA, 20 min, on ice), MeOH (15 min, −20 °C), 0.3% Triton-X100 (10 min, RT) in CMF-PBS, prior to blocking (1 h, RT) in 10% FBS, 0.05% Tween-20, and 1.25% Human BD Fc Block™ (BD Biosciences, San Jose, CA). The samples were then incubated with a first set of primary (1:200, 1 h, RT, in blocking buffer), and secondary (1:1000, 1 h, RT) antibodies, and the second set of primary (1:200, 30 min, RT) and secondary (1:1000, 30 min, RT) antibodies (in blocking buffer). Samples were washed in between all antibody steps (3x, 700 x g, 5 min). Primary antibodies were mouse anti-C15-VP2 (MedImmune, Gaithersburg, MD), mouse anti-FLJ23834 (anti-CDHR3), rabbit anti-acetylated-alpha-tubulin, rabbit anti-Muc5AC, mouse IgG1 isotype, and mouse IgG2b isotype (AbCam, Cambridge, MA). Secondary antibodies (Alexa Fluor 350, Alexa Fluor 568, Alexa Fluor 647) and wheat germ agglutinin (Alexa Fluor 350-conjugated) were from Life Technologies (Grand Island, NY). Data from labelled cells were acquired on a Fortessa (BD Biosciences) that was calibrated using Rainbow Fluorescent Particles (RFP-30-5A, Spherotech, Lake Forest, IL) and analyzed with FlowJo software version 10 (Tree Star, San Carlos, CA). For analysis, we normalized our median fluorescence intensity of CDHR3 (MFI_CDHR3_) data to the double-negative (nonciliated, CDHR3-) population in each experiment to obtain the relative MFI_CDHR3_ (rMFI_CDHR3_).

### Western blot

ALI cells were lysed with 2X SDS buffer and proteins were denatured by boiling at 95 °C for 5 min. Then, 15 μL of protein samples were loaded onto mini-Protean TGX gels and protein bands were transferred to PVDF membrane and blocked with 3% non-fat dry milk in TBST. Primary and secondary antibodies were as follows: anti-CDHR3 polyclonal antibody (1:1000, Sigma HPA011218) and anti-rabbit IgG-peroxidase (Sigma A6154, 1:5000) and the substract was SuperSignal West Femto Maximum Sensitivity chemiluminescent substrate (Thermo Scientific, 34095).

### Statistics

Data were analyzed using SigmaPlot version 11.0 (Systat Software, Inc., San Jose, CA). One-way Repeated Measures ANOVAs were used to compare three or more groups, and square-root-transformed data was used to analyze data from PneumaCult™-differentiated cultures.

## Results

### RV-C15 infection of HBEC-ALI cultures result in diffuse, apical shedding of intact cells

**Fig. 1:**
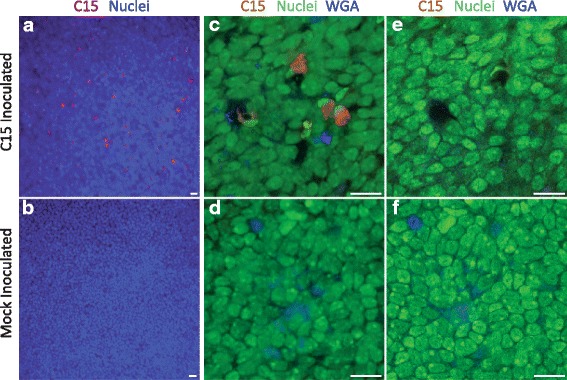
C15 inoculation of airway epithelial cells causes a speckled pattern of infection and infected cell shedding. HBEC-ALI cultures were inoculated for 18 h with C15 or media alone and imaged by fluorescent microscopy (**a** and **b**, respectively). Nuclei stained with Hoechst (*blue*), C15 capsid stained with monoclonal antibody against VP2 (*red*). Inoculated cultures were also imaged by confocal microscopy and analyzed by z-stacking (**c** and **d**) or apical surface views (**e** and **f**). Nuclei stained with Syto-13 (*green*), secretory cells stained with WGA (*blue*), and C15 capsid stained with monoclonal antibody (*orange*). Scale bars indicate 20 μm

To visualize RV-C-infected cells, human bronchial epithelial cells (HBECs) were differentiated in vitro at an air-liquid interface (ALI) for 30–50 days, and then inoculated with RV-C15 (C15). After 16–18 h, immunofluorescent staining revealed cells with bright cytoplasmic staining for the viral capsid. These C15-positive (C15+) cells were distributed diffusely along the epithelium (Fig. 1a). Virus-infected cells often appeared rounded, and the brightest C15+ cells were observed above the epithelial layer among the epithelial cilia. Mock-inoculated cultures demonstrated a uniform, undisrupted epithelium (Fig. 1b).

Following inoculation, cell-sized holes in the epithelial layer were noted by confocal microscopy just basal to C15+ cells that were not observed in mock-inoculated cultures (Fig. 1c-f). In order to determine whether these holes in the epithelium were created by shedding of infected cells, we inoculated HBEC-ALI cultures with a C15 genetically engineered to express GFP during viral replication (C15-GFP) and performed time-lapse fluorescent imaging over the course of 30 h of viral infection (Additional file 1). Cells expressing GFP indeed rounded in place and then detached from the epithelial layer, leaving gaps in the epithelium that subsequently contracted over the course of the experiment.

### Ciliated cells are the host cell for C15

**Fig. 2:**
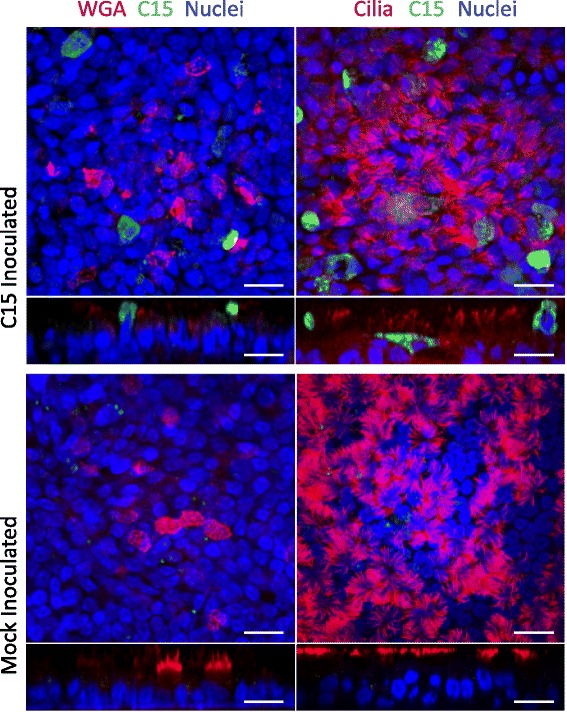
Analysis of C15 infectivity by immunofluorescent staining. HBEC-ALI cultures were infected for 18 h with C15 and imaged apically and z-stacked orthogonally by confocal microscopy. Secretory and ciliated cells were stained with WGA and rabbit polyclonal antibody against aat, respectively (*false color red*), C15 capsid stained with monoclonal antibody (*false color green*), nuclei stained with Syto-13 (*false color blue*). Scale bars indicate 20 μm

We next assessed by immunofluorescence the colocalization of C15 staining together with markers of secretory cells (wheat germ agglutinin [WGA]) and ciliated cells (acetylated-alpha-tubulin [aat]), respectively (Fig. 2). Many C15+ cells had aat staining of cilia on the apical membrane. On the other hand, cells staining positive for both C15 and WGA were not observed.

**Fig. 3:**
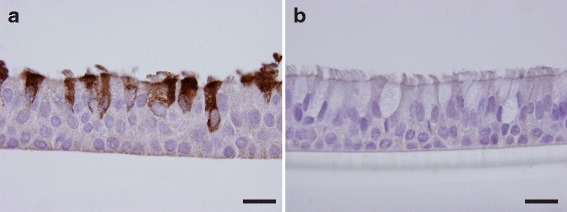
Immunohistochemical analysis of C15 infectivity in HBEC-ALI cultures. PCM-differentiated HBEC-ALI cultures were inoculated with **a** C15 or **b** media alone, stained for C15 capsid, and imaged by light microscopy. C15 capsid is represented by *brown-staining cells*. Scale bars indicate 20 μm

To define the morphology of infected cells, HBEC-ALI cultures infected for 10 h were analyzed by immunohistochemistry (IHC, Fig. 3a). Again, the vast majority of C15+ cells possessed visible cilia with C15 staining observed primarily in the cytoplasm of ciliated cells. Interestingly, the cilia of C15+ cells also stained positive for C15 capsid protein, while the cilia of adjacent, uninfected cells did not. Mock inoculated cultures demonstrated intact respiratory epithelium with a diverse range of recognizable, ciliated, goblet, and basal cell types (Fig. 3b).

**Fig. 4:**
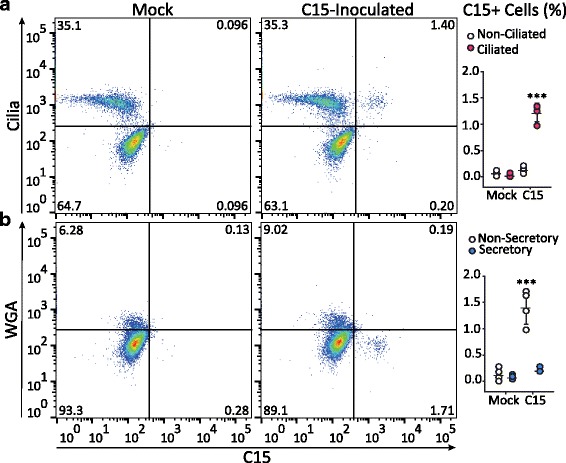
Single cell analysis of RV-C infection and epithelial cell surface markers. HBEC-ALI cultures infected or mock-infected for 18 h with C15 or BEGM alone, respectively, were labeled with antibodies against C15 capsid (C15), and either **a** acetylated-alpha-tubulin (aat, Cilia) or **b** fluorescently labeled-WGA (WGA) and analyzed by flow cytometry. The graphs summarize the percentage of C15+ cells in ciliated and non-ciliated cells (*n* = 4 independent experiments, two cell donors). Mock, mock-inoculated cultures; C15, C15-inoculated cultures. *** *p* < 0.001

Next, we performed a single cell analysis of C15 infection with markers of airway epithelial cell subsets. HBEC cultures differentiated using BEGM were inoculated (18 h) with C15, and then single cell suspensions were analyzed for markers of ciliated or secretory phenotype (Fig. 4). We consistently observed low frequencies of C15 infection (1.0 to 1.4% C15+ cells). Inoculation caused increased staining for C15 in ciliated cells (*p* < 0.001, Fig. 4a), but not non-ciliated cells (*p* = ns). Analysis of cells stained with WGA further confirmed that secretory cells were not infected with C15 (*p* = ns, Fig. 4b).

### Highly ciliated cultures are more susceptible to C15 infection

**Fig. 5:**
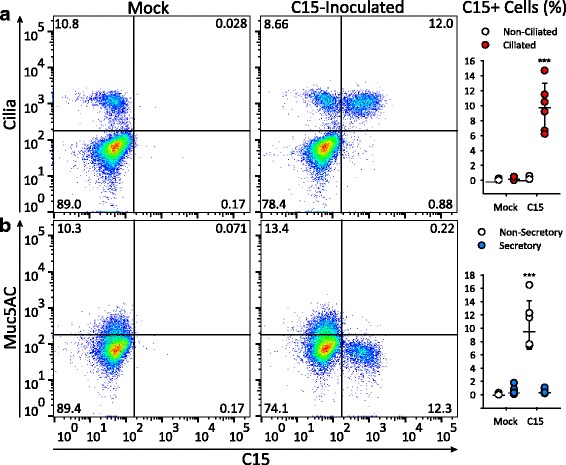
Highly differentiated cells are more susceptible to RV-C infection. Differentiated cultures were incubated for 18 h after inoculation with C15 or BEGM alone, labeled with antibodies against C15 capsid (C15) and either **a** aat (Cilia) or **b** Muc5AC and analyzed by flow cytometry. The graphs show the percentage of C15-positive cells by cell type (*n* = 6). *** *p* < 0.001

We consistently observed that highly ciliated cell cultures were more susceptible to RV-C infection [40, 42]. Preliminary experiments revealed that cells cultured using a different HBEC culture medium (PneumaCult™, Stem Cell Technologies, Vancouver, Canada) had more plentiful ciliated cells [43]. We hypothesized that the greater differentiation of ciliated cells achieved using PneumaCult™ media (PCM) would lead to greater susceptibility to RV-C infection. Thus, we inoculated HBEC-ALI cultures for 18 h with either C15 or BEGM alone, and then analyzed single cell suspensions for markers of epithelial cell subsets and C15 infection (Fig. 5). When compared to BEGM-differentiated cultures, the proportion of ciliated cells was lower in PCM-differentiated cultures (*p* ≤ 0.001, Additional file 2: Figure S1A). However, C15 staining was observed in 6.2 to 14.7% of HBECs differentiated in PCM, which represented a 5-10-fold increased frequency of infected cells compared to cells differentiated in BEGM (Fig. 5a). Interestingly, following C15 inoculation the proportion of ciliated cells was 13.0% greater compared to mock-inoculated cultures (*p* ≤ 0.01, Additional file 2: Figure S1B). Again, C15 replicated only in ciliated cells (*p* < 0.001, Fig. 5a), as there was no significant C15+ staining of non-ciliated cells or Muc5AC+ cells compared to mock-inoculated cells (*p* = ns, Fig. 5b).

### Ciliated bronchial epithelial cells express the highest levels of CDHR3

**Fig. 6:**
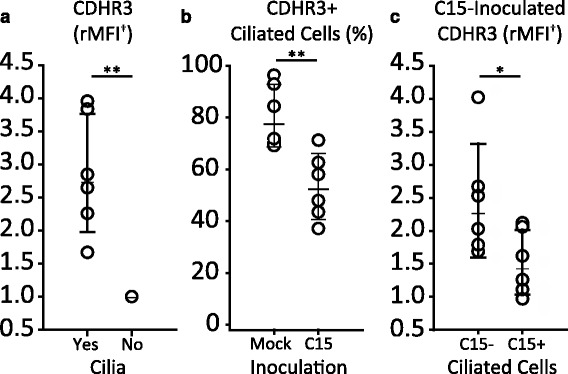
CDHR3 is predominantly expressed by ciliated epithelial cells and diminishes following C15 inoculation. PC-differentiated cultures inoculated or mock-inoculated for 18 h with C15 (C15+) or BEGM alone (Mock), respectively, were labeled with antibodies against C15 capsid (C15), CDHR3, and aat (cilia) and analyzed by flow cytometry (*n* = 6). **a** Relative median fluorescence intensity (rMFI) of CDHR3 in ciliated (Yes) and nonciliated (No) cell populations of mock-inoculated cultures. **b** Frequency of CDHR3 positive ciliated cells out of the total ciliated cell population in mock- and C15-inoculated cultures. **c** rMFI of CDHR3 in ciliated cells negative (C15-) or positive (C15+) for C15 staining. Values are normalized to the double-negative (nonciliated and CDHR3-) population in each experiment. ** *p* ≤ 0.01, * *p* ≤ 0.05

We recently demonstrated that surface expression of cadherin-related family member 3 (CDHR3) enabled RV-C entry into normally non-permissive HeLa cells followed by viral replication [38]. Replication of C15 in ciliated cells suggested that CDHR3 expression might be restricted to these cells. To test this hypothesis, we inoculated differentiated HBECs with either C15 or medium alone and stained cells for cilia, C15, and CDHR3 (Fig. 6). In mock infected cultures, the staining of CDHR3 was 2.87-times brighter on ciliated cells compared to nonciliated cells (*p* ≤ 0.01, Fig. 6a). Furthermore, C15 inoculation reduced CDHR3-positive staining on ciliated cells from 80.8% to 53.4% (*p* ≤ 0.01, Fig. 6b). Similarly, C15 inoculation reduced the intensity of CDHR3 staining of ciliated cells by about one third (Fig. 6c).

### RV-C binding and replication positively correlates with CDHR3 expression in differentiating human bronchial epithelial cells

**Fig. 7:**
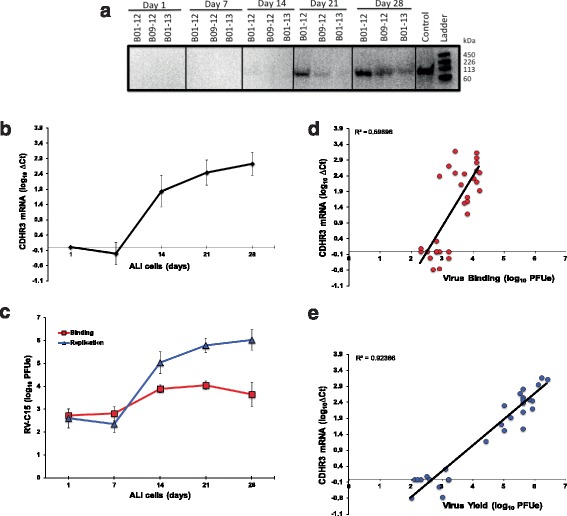
CDHR3 expression in differentiating bronchial epithelial cells positively correlates with RV-C binding and replication. **a** CDHR3 protein expression (~100 kDa) and **b** mRNA expression (*n* = 8) by differentiating airway epithelial cells in ALI using PCM from day 1 to day 28. **c** BECs in ALI were infected weekly with C15 and the infected cell samples were collected and analyzed for viral binding (2 hpi) and viral yield (24 hpi). **d** and **e** Correlations (linear regression) between cellular CDHR3 mRNA expression and C15 binding and yield

We next tested whether the level of CDHR3 expression in host cells correlated with RV-C cell binding and subsequent virus replication. To test this hypothesis, we cultured 8 HBEC-ALI cultures in PCM to allow differentiation over a 28-day time period and monitored CDHR3 mRNA and protein expression at weekly intervals. In addition, each week, aliquots of differentiating cells were inoculated with C15 (2x10^6^ PFU equivalents) and cell lysates were collected at 2 and 24 h post inoculation (hpi) to test for C15 binding and replication, respectively. Cellular expression of CDHR3 mRNA and protein in differentiating HBECs increased through day 28 (Fig. 7a-b). Congruently, C15 binding and replication titers increased over time in the differentiating human BECs (Fig. 7c). CDHR3 mRNA expression strongly correlated with C15 binding (r = +0.77, Fig. 7d) and C15 replication (r = +0.96, Fig. 7e). The largest increase in CDHR3 mRNA expression was observed from day 7 to day 14, which corresponded with large increases in C15 binding and replication.

## Discussion

Since their discovery in 2006, the RV-C have been identified as important contributors to severe respiratory illness in infants and exacerbations of chronic airway disease, yet little is known about how they interact with airway cells. Here, we demonstrated that among epithelial cells, CDHR3 protein expression in ALI cells is developmentally regulated and largely confined to ciliated cells. Several techniques were used to show that RV-C replicate in ciliated AECs, but not secretory cells. Finally, during the differentiation process, RV-C binding and especially replication were closely correlated to temporal patterns of CDHR3 induction. These findings show evidence of a close relationship between ciliated epithelial cells, CDHR3 expression, and susceptibility to RV-C infection.

Ciliated AECs are targets for multiple RNA viruses (e.g. avian influenza virus, coronavirus, RSV, parainfluenza virus, and RV-A), and infections thereof have been correlated to viral receptor expression [22, 27, 28, 35, 44–49]. CDHR3 genotype was initially linked to the risk of childhood asthma with acute severe exacerbations, and subsequent studies showed that transfection of HeLa cells with CDHR3 rendered the cells permissive for RV-C binding and replication [50]. Furthermore, the asthma-risk genotype of CDHR3 conferred increased RV-C binding and replication [38]. Our data extend observations into primary AEC, and demonstrate that CDHR3 expression was high and largely restricted to ciliated cells. Notably, RV-C does not infect secretory cells, which can be overrepresented in chronically inflamed airways [35, 51]. These findings were consistent for cells differentiated in two different culture media (BEGM and PCM). We found that BEGM-cultures have larger proportions of ciliated cells, but only a small fraction of these cells could be infected with RV-C. Cultures differentiated in PCM had fewer ciliated cells, together with an increased representation of other differentiated cell types (goblet, clara, basal, etc.), more closely mimicking airway biology [43]. A larger fraction of ciliated cells cultured in PCM could be infected with RV-C, however, the majority of ciliated cells were not infected in either culture system, despite an excess of virus. This finding may indicate the presence of unidentified cofactors or specific cellular properties required for RV-C binding or entry. Many viruses either utilize co-receptors or have cofactors that facilitate cellular binding. Poliovirus, for example, utilizes bacterial polysaccharides to increase the kinetics of binding to its host cells in the gut [52, 53]. Alternately, some cells may be protected from RV-C infection by overlying mucus, or perhaps CDHR3 exposure to RV-C is reduced by sequestration to lateral membranes (a common location for intracellular cadherins) [54].

RV-C infection led to changes in cell composition, including a reduction in CDHR3 expression and increased percentage of ciliated cells. The reduction in CDHR3 staining could be due to viral shutoff of host protein synthesis, CDHR3 down-regulation following internalization of the virus/receptor complex, or lysis of infected cells. The apparent increase in ciliated cells following C15-inoculation may occur through infection-induced differentiation from basal cells, or perhaps through transdifferentiation of goblet or clara cells to ciliated cells, a concept proposed in previous studies of airway epithelia and infection [55].

While our data indicate that CDHR3 expression is necessary for RV-C susceptibility in primary airway epithelial cells, other factors may promote or inhibit RV binding or entry. For example, A16 has been shown to primarily target ciliated cells, even though ICAM-1 is also expressed on some nonciliated cells [35]. Notably, virus-sized (40 nm) nanoparticles adhere to motile cilia in vitro, demonstrating a potential nonspecific mechanism for viral adhesion to ciliated cells [56]. These findings raise the possibility that RV may adhere to cilia to facilitate cell entry, and this is consistent with our observations that cilia of infected cells stained positive for C15 (Fig. 3a).

The speckled pattern of C15 infection of AECs is consistent with what has been observed for A16 [35]. The shedding of infected cells, perhaps before the virus is able to spread to surrounding cells, could contribute to the patchy distribution of infection. The resulting gaps in the epithelium would likely impair barrier function and perhaps facilitate bacterial superinfection [2, 4, 5, 7]. These effects have been noted following influenza A infections in vitro [57]. It will be of interest in future studies to determine whether CDHR3 genotype or disease states influence patterns of CDHR3 expression and RV-C infection.

Strengths of these studies included the use of viruses that were cloned from clinical isolates, and the use of primary cultures of airway epithelial cells differentiated at ALI. There are also limitations that should be considered in interpreting these findings. First, the only available anti-CDHR3 monoclonal antibody binds to an intracellular epitope, which prevented a discrete quantification of CDHR3 displayed on the cell surface, where it is accessible to virus. Secondly, our experiments involved cultured cells, and additional studies are needed to map the distribution of RV-C infection in vivo.

## Conclusions

Our findings establish that RV-C primarily infects ciliated AECs, and that expression of CDHR3 is largely restricted to these cells. These findings provide a foundation for studying RV-C entry mechanisms into their primary host cell, establish a robust in vitro model of RV-C infection, and suggest that factors that regulate CDHR3 expression or differentiation of ciliated cells may influence susceptibility to RV-C infection in vivo. These models will enable future studies to fill crucial knowledge gaps in the pathogenesis of this clinically important RV species.

## Additional files

Additional file 1: C15-infected cells are shed from intact respiratory epithelium in vitro. HBEC-ALI cultures were infected for 18 h with C15 that expresses GFP during replication (C15-GFP, green) and fluorescently (Left, GFP video) or phase-contrast (Right, grayscale video) imaged every 10–15 min for 30 h. Notably shed cells are labeled in the first frame (black arrows). Original magnification: 20x. (ZIP 13284 kb)

Additional file 2: Figure S1. Ciliated cells are underrepresented in PCM-cultures compared to BEGM-differentiated cultures, but dramatically increase following C15 inoculation. Differentiated cultures were incubated for 18 h after inoculation with C15 or BEGM alone, labeled with antibodies against C15 capsid and aat and analyzed by flow cytometry. Figure compares the percentage of ciliated cells out of all cells analyzed of (A) BEGM (*n* = 4) and PCM-differentiated cultures (*n* = 6), and (B) mock- and C15-inoculated PCM-differentiated cultures (*n* = 6). *** *p* < 0.001, ** *p* < 0.01. (PDF 86.5 kb)

## Abbreviations

A16: RV-A16
Aat: acetylated-alpha-tubulin
AECs: airway epithelial cells
ALI: air-liquid interface
C15: RV-C15
CDHR3: cadherin related family member 3
COPD: chronic obstructive pulmonary disease
HBEC: human bronchial epithelial cells
IHC: Immunohistochemistry
MFI-CDHR3: Median fluorescence intensity of CDHR3
PCM: PneumaCult(TM) media
PFA: paraformaldehyde
PFUe: plaque forming unit equivalents
rMFI-CDHR3: Relative MFI-CDHR3
RT: room temperature
RV: rhinovirus
RV-C: rhinovirus C
WGA: wheat germ agglutinin

## References

[1] Fendrick AM, Monto AS, Nightengale B, Sarnes M (2003). The economic burden of non-influenza-related viral respiratory tract infection in the United States. Arch Intern Med.

[2] Jakab GJ (1985). Mechanisms of bacterial superinfections in viral pneumonias. Schweiz Med Wochenschr.

[3] Monto AS, Bryan ER, Ohmit S (1987). Rhinovirus infections in Tecumseh, Michigan: frequency of illness and number of serotypes. J Infect Dis.

[4] Hament J-M, Kimpen JL, Fleer A, Wolfs TF (1999). Respiratory viral infection predisposing for bacterial disease: a concise review. FEMS Immunol Med Microbiol.

[5] Ishizuka S, Yamaya M, Suzuki T, Takahashi H, Ida S, Sasaki T (2003). Effects of rhinovirus infection on the adherence of Streptococcus pneumoniae to cultured human airway epithelial cells. J Infect Dis.

[6] Monto AS (2003). Epidemiology of viral respiratory infections. Dis Mon.

[7] Bosch AATM, Biesbroek G, Trzcinski K, Sanders EAM, Bogaert D (2013). Viral and Bacterial Interactions in the Upper Respiratory Tract. PLoS Pathog.

[8] Johnston SL, Pattemore PK, Sanderson G, Smith S, Lampe F, Josephs L (1995). Community study of role of viral infections in exacerbations of asthma in 9–11 year old children. BMJ.

[9] Friedlander SL, Busse WW (2005). The role of rhinovirus in asthma exacerbations. J Allergy Clin Immunol.

[10] Miller EK, Lu X, Erdman DD, Poehling KA, Zhu Y, Griffin MR (2007). Rhinovirus-associated hospitalizations in young children. J Infect Dis.

[11] Brownlee JW, Turner RB (2008). New developments in the epidemiology and clinical spectrum of rhinovirus infections. Curr Opin Pediatr.

[12] Peltola V, Waris M, Osterback R, Susi P, Ruuskanen O, Hyypiä T (2008). Rhinovirus transmission within families with children: incidence of symptomatic and asymptomatic infections. J Infect Dis.

[13] Gern JE (2010). The ABCs of rhinoviruses, wheezing, and asthma. J Virol.

[14] Cox DW, Bizzintino J, Ferrari G, Khoo SK, Zhang G, Whelan S (2013). Human rhinovirus species C infection in young children with acute wheeze is associated with increased acute respiratory hospital admissions. Am J Respir Crit Care Med.

[15] Gern JE, Busse WW (2002). Relationship of viral infections to wheezing illnesses and asthma. Nat Rev Immunol.

[16] Lemanske RF, Jackson DJ, Gangnon RE, Evans MD, Li Z, Shult PA (2005). Rhinovirus illnesses during infancy predict subsequent childhood wheezing. J Allergy Clin Immunol.

[17] Jackson DJ, Gangnon RE, Evans MD, Roberg KA, Anderson EL, Pappas TE (2008). Wheezing rhinovirus illnesses in early life predict asthma development in high-risk children. Am J Respir Crit Care Med.

[18] Walton RP, Johnston SL (2008). Role of respiratory viral infections in the development of atopic conditions. Curr Opin Allergy Clin Immunol.

[19] Jackson DJ, Evans MD, Gangnon RE, Tisler CJ, Pappas TE, Lee W-M (2012). Evidence for a Causal Relationship between Allergic Sensitization and Rhinovirus Wheezing in Early Life. Am J Respir Crit Care Med.

[20] Lee W-M, Lemanske RF, Evans MD, Vang F, Pappas T, Gangnon R (2012). Human rhinovirus species and season of infection determine illness severity. Am J Respir Crit Care Med.

[21] Nakagome K, Bochkov YA, Ashraf S, Brockman-Schneider RA, Evans MD, Pasic TR (2014). Effects of rhinovirus species on viral replication and cytokine production. J Allergy Clin Immunol.

[22] Matrosovich MN, Matrosovich TY, Gray T, Roberts NA, Klenk H-D (2004). Human and avian influenza viruses target different cell types in cultures of human airway epithelium. Proc Natl Acad Sci U S A.

[23] Mounts AW, Kwong H, Izurieta HS, Ho Y, Au T, Lee M (1999). Case–control study of risk factors for avian influenza A (H5N1) disease, Hong Kong, 1997. J Infect Dis.

[24] Centers for Disease Control and Prevention (CDC) (2003). Update: influenza activity--United States and worldwide, 2002–03 season, and composition of the 2003–04 influenza vaccine. MMWR Morb Mortal Wkly Rep.

[25] Matrosovich M, Tuzikov A, Bovin N, Gambaryan A, Klimov A, Castrucci MR (2000). Early alterations of the receptor-binding properties of H1, H2, and H3 avian influenza virus hemagglutinins after their introduction into mammals. J Virol.

[26] Matrosovich M, Matrosovich T, Uhlendorff J, Garten W, Klenk H-D (2007). Avian-virus-like receptor specificity of the hemagglutinin impedes influenza virus replication in cultures of human airway epithelium. Virology.

[27] Sims AC, Baric RS, Yount B, Burkett SE, Collins PL, Pickles RJ (2005). Severe acute respiratory syndrome coronavirus infection of human ciliated airway epithelia: role of ciliated cells in viral spread in the conducting airways of the lungs. J Virol.

[28] Chan RWY, Chan MCW, Agnihothram S, Chan LLY, Kuok DIT, Fong JHM (2013). Tropism of and innate immune responses to the novel human betacoronavirus lineage C virus in human ex vivo respiratory organ cultures. J Virol.

[29] To KF, Tong JHM, Chan PKS, Au FWL, Chim SSC, Chan KCA (2004). Tissue and cellular tropism of the coronavirus associated with severe acute respiratory syndrome: an in-situ hybridization study of fatal cases. J Pathol.

[30] Matsuyama S, Nagata N, Shirato K, Kawase M, Takeda M, Taguchi F (2010). Efficient Activation of the Severe Acute Respiratory Syndrome Coronavirus Spike Protein by the Transmembrane Protease TMPRSS2. J Virol.

[31] de Arruda E, Mifflin TE, Gwaltney JM, Winther B, Hayden FG (1991). Localization of rhinovirus replication in vitro with in situ hybridization. J Med Virol.

[32] Winther B, Greve JM, Gwaltney JM, Innes DJ, Eastham JR, McClelland A (1997). Surface expression of intercellular adhesion molecule 1 on epithelial cells in the human adenoid. J Infect Dis.

[33] Jakiela B, Brockman-Schneider R, Amineva S, Lee W-M, Gern JE (2008). Basal cells of differentiated bronchial epithelium are more susceptible to rhinovirus infection. Am J Respir Cell Mol Biol.

[34] Lachowicz-Scroggins ME, Boushey HA, Finkbeiner WE, Widdicombe JH (2010). Interleukin-13-induced mucous metaplasia increases susceptibility of human airway epithelium to rhinovirus infection. Am J Respir Cell Mol Biol.

[35] Jakiela B, Gielicz A, Plutecka H, Hubalewska-Mazgaj M, Mastalerz L, Bochenek G (2014). Th2-type cytokine-induced mucus metaplasia decreases susceptibility of human bronchial epithelium to rhinovirus infection. Am J Respir Cell Mol Biol.

[36] Zhu J, Rogers AV, Burke-Gaffney A, Hellewell PG, Jeffery PK (1999). Cytokine-induced airway epithelial ICAM-1 upregulation: quantification by high-resolution scanning and transmission electron microscopy. Eur Respir J.

[37] Bochkov YA, Palmenberg AC, Lee W-M, Rathe JA, Amineva SP, Sun X (2011). Molecular modeling, organ culture and reverse genetics for a newly identified human rhinovirus C. Nat Med.

[38] Bochkov YA, Watters K, Ashraf S, Griggs TF, Devries MK, Jackson DJ (2015). Cadherin-related family member 3, a childhood asthma susceptibility gene product, mediates rhinovirus C binding and replication. Proc Natl Acad Sci U S A.

[39] Griggs TF, Bochkov YA, Nakagome K, Palmenberg AC, Gern JE (2015). Production, purification, and capsid stability of rhinovirus C types. J Virol Methods.

[40] Ashraf S, Brockman-Schneider R, Bochkov YA, Pasic TR, Gern JE (2013). Biological characteristics and propagation of human rhinovirus-C in differentiated sinus epithelial cells. Virology.

[41] Hao W, Bernard K, Patel N, Ulbrandt N, Feng H, Svabek C (2012). Infection and propagation of human rhinovirus C in human airway epithelial cells. J Virol.

[42] Bochkov YA, Gern JE (2012). Clinical and molecular features of human rhinovirus C. Microbes Infect Inst Pasteur.

[43] Wadsworth SJ, Riedel M, Afshari AE, Louis S, Dorscheid D (2012). PneumaCultTM-ALI: an improved media for mucociliary differentiation of primary human bronchial epithelial cells. Am J Respir Crit Care Med.

[44] Villenave R, Shields MD, Power UF (2013). Respiratory syncytial virus interaction with human airway epithelium. Trends Microbiol.

[45] Zhang L, Peeples ME, Boucher RC, Collins PL, Pickles RJ (2002). Respiratory syncytial virus infection of human airway epithelial cells is polarized, specific to ciliated cells, and without obvious cytopathology. J Virol.

[46] Zhang L, Collins PL, Lamb RA, Pickles RJ (2011). Comparison of differing cytopathic effects in human airway epithelium of parainfluenza virus 5 (W3A), parainfluenza virus type 3, and respiratory syncytial virus. Virology.

[47] Smith CM, Kulkarni H, Radhakrishnan P, Rutman A, Bankart MJ, Williams G (2014). Ciliary dyskinesia is an early feature of respiratory syncytial virus infection. Eur Respir J.

[48] Mata M, Sarrion I, Armengot M, Carda C, Martinez I, Melero JA (2012). Respiratory syncytial virus inhibits ciliagenesis in differentiated normal human bronchial epithelial cells: effectiveness of N-acetylcysteine. PloS One.

[49] Villenave R, Thavagnanam S, Sarlang S, Parker J, Douglas I, Skibinski G (2012). In vitro modeling of respiratory syncytial virus infection of pediatric bronchial epithelium, the primary target of infection in vivo. Proc Natl Acad Sci U S A.

[50] Bønnelykke K, Sleiman P, Nielsen K, Kreiner-Møller E, Mercader JM, Belgrave D (2014). A genome-wide association study identifies CDHR3 as a susceptibility locus for early childhood asthma with severe exacerbations. Nat Genet.

[51] Tesfaigzi Y (2008). Regulation of mucous cell metaplasia in bronchial asthma. Curr Mol Med.

[52] Kuss SK, Best GT, Etheredge CA, Pruijssers AJ, Frierson JM, Hooper LV (2011). Intestinal microbiota promote enteric virus replication and systemic pathogenesis. Science.

[53] Robinson CM, Jesudhasan PR, Pfeiffer JK (2014). Bacterial lipopolysaccharide binding enhances virion stability and promotes environmental fitness of an enteric virus. Cell Host Microbe.

[54] Nawijn MC, Hackett TL, Postma DS, van Oosterhout AJM, Heijink IH (2011). E-cadherin: gatekeeper of airway mucosa and allergic sensitization. Trends Immunol.

[55] Patel AC, Brody SL, Stappenbeck TS, Holtzman MJ (2011). Tracking cell lineage to rediscover (again) the switch from ciliated to mucous cells. Am J Respir Cell Mol Biol.

[56] Bermbach S, Weinhold K, Roeder T, Petersen F, Kugler C, Goldmann T (2014). Mechanisms of Cilia-Driven Transport in the Airways in the Absence of Mucus. Am J Respir Cell Mol Biol.

[57] Sajjan U, Wang Q, Zhao Y, Gruenert DC, Hershenson MB (2008). Rhinovirus Disrupts the Barrier Function of Polarized Airway Epithelial Cells. Am J Respir Crit Care Med.

