# Supplementary material for: Rhinovirus C targets ciliated airway epithelial cells
Source: Respir Res. 2017 May 4;18:84. doi: 10.1186/s12931-017-0567-0 (PMC5418766; doi:10.1186/s12931-017-0567-0)
Supplement: Supplementary file 2 — Ciliated cells are underrepresented in PCM-cultures compared to BEGM-differentiated cultures, but dramatically increase following C15 inoculation. Differentiated cultures were incubated for 18 h after inoculation with C15 or BEGM alone, labeled with antibodies against C15 capsid and aat and analyzed by flow cytometry. Figure compares the percentage of ciliated cells out of all cells analyzed of (A) BEGM (n = 4) and PCM-differentiated cultures (n = 6), and (B) mock- and C15-inoculated PCM-differentiated cultures (n = 6). *** p < 0.001, ** p < 0.01. (PDF 86.5 kb) [file 12931_2017_567_MOESM2_ESM.pdf]

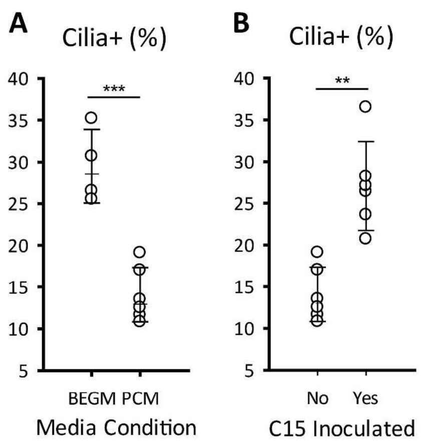

Ciliated cells are underrepresented in PCM-cultures compared to BEGM-differentiated cultures, but dramatically increase following C15 inoculation. Differentiated cultures were incubated for 18h after inoculation with C15 or BEGM alone, labeled with antibodies against C15 capsid and aat and analyzed by flow cytometry. Figure compares the percentage of ciliated cells out of all cells analyzed of (A) BEGM (n=4) and PCM-differentiated cultures (n=6), and (B) mock- and C15-inoculated PCM-differentiated cultures (n=6). *** $p<0.001$, ** $p<0.01$.

Supplemental Figure 1

279x361mm (300 x 300 DPI)
